# Metagenome-assembled genomes reveal greatly expanded taxonomic and functional diversification of the abundant marine *Roseobacter* RCA cluster

**DOI:** 10.1186/s40168-023-01644-5

**Published:** 2023-11-25

**Authors:** Yanting Liu, Thorsten Brinkhoff, Martine Berger, Anja Poehlein, Sonja Voget, Lucas Paoli, Shinichi Sunagawa, Rudolf Amann, Meinhard Simon

**Affiliations:** 1 Institute for Chemistry and Biology of the Marine Environment, University of Oldenburg, Carl Von Ossietzky Str. 9-11, 26129 Oldenburg, Germany; 2 Max Planck Institute for Marine Microbiology, Bremen, Germany; 3 State Key Laboratory for Marine Environmental Science, Institute of Marine Microbes and Ecospheres, Xiamen University, Xiamen, People’s Republic of China; 4 Department of Genomic and Applied Microbiology & Göttingen Genomics Laboratory, Georg-August University Göttingen, Grisebachstr. 8, 37077 Göttingen, Germany; 5 Department of Biology, Institute of Microbiology and Swiss Institute of Bioinformatics, ETH Zürich, Zurich, Switzerland; 6 Helmholtz Institute for Functional Marine Biodiversity at the University of Oldenburg (HIFMB), Ammerländer Heerstr. 231, 26129 Oldenburg, Germany

**Keywords:** RCA cluster, *Roseobacteraceae*, *Rhodobacteraceae*, Metagenome-assembled genomes, SeqCode, Phylogenomics, Proteorhodopsin, Horizontal gene transfer, Genome streamlining, Genome content

## Abstract

**Background:**

The RCA (*Roseobacter* clade affiliated) cluster belongs to the family *Roseobacteracea* and represents a major *Roseobacter* lineage in temperate to polar oceans. Despite its prevalence and abundance, only a few genomes and one described species, *Planktomarina temperata*, exist. To gain more insights into our limited understanding of this cluster and its taxonomic and functional diversity and biogeography, we screened metagenomic datasets from the global oceans and reconstructed metagenome-assembled genomes (MAG) affiliated to this cluster.

**Results:**

The total of 82 MAGs, plus five genomes of isolates, reveal an unexpected diversity and novel insights into the genomic features, the functional diversity, and greatly refined biogeographic patterns of the RCA cluster. This cluster is subdivided into three genera: *Planktomarina*, *Pseudoplanktomarina*, and the most deeply branching *Candidatus* Paraplanktomarina. Six of the eight *Planktomarina* species have larger genome sizes (2.44–3.12 Mbp) and higher G + C contents (46.36–53.70%) than the four *Pseudoplanktomarina* species (2.26–2.72 Mbp, 42.22–43.72 G + C%). *Cand*. Paraplanktomarina is represented only by one species with a genome size of 2.40 Mbp and a G + C content of 45.85%. Three novel species of the genera *Planktomarina* and *Pseudoplanktomarina* are validly described according to the SeqCode nomenclature for prokaryotic genomes. Aerobic anoxygenic photosynthesis (AAP) is encoded in three *Planktomarina* species. Unexpectedly, proteorhodopsin (PR) is encoded in the other *Planktomarina* and all *Pseudoplanktomarina* species, suggesting that this light-driven proton pump is the most important mode of acquiring complementary energy of the RCA cluster. The *Pseudoplanktomarina* species exhibit differences in functional traits compared to *Planktomarina* species and adaptations to more resource-limited conditions. An assessment of the global biogeography of the different species greatly expands the range of occurrence and shows that the different species exhibit distinct biogeographic patterns. They partially reflect the genomic features of the species.

**Conclusions:**

Our detailed MAG-based analyses shed new light on the diversification, environmental adaptation, and global biogeography of a major lineage of pelagic bacteria. The taxonomic delineation and validation by the SeqCode nomenclature of prominent genera and species of the RCA cluster may be a promising way for a refined taxonomic identification of major prokaryotic lineages and sublineages in marine and other prokaryotic communities assessed by metagenomics approaches.

Video Abstract

**Supplementary Information:**

The online version contains supplementary material available at 10.1186/s40168-023-01644-5.

## Introduction

The RCA (*Roseobacter* clade affiliated) cluster is one of the largest lineages of the *Roseobacter* group, recently reclassified as family *Roseobacteraceae* of *Rhodobacterales* [1]. Its abundance, distribution, and ecological significance in marine ecosystems have been investigated in the past 20 years [2–7]. Members of this cluster are widely distributed from temperate to polar regions of the global oceans and constitute ~ 10 to 35% of the total bacterial communities [3, 4, 8] but are absent from permanently stratified (sub)tropical regions [2–4, 8, 9]. The RCA cluster often dominates the *Roseobacter* group in marine pelagic systems [10–13], is closely associated with phytoplankton blooms, and is a major player in processing phytoplankton-derived organic matter [5, 6, 14, 15]. The 16S rRNA gene sequence similarity within the cluster is > 98% [6], but the further taxonomic and genomic substructure of the RCA cluster is unknown due to the few isolates and genomes available [16, 17].

The primary genome characteristics of the RCA cluster were previously analyzed in the type strain, *Planktomarina temperata* RCA23, isolated from the North Sea [6]. In addition to many functional genomic features typical for the *Roseobacter* group, this strain has a relatively small genome, encodes various modes of complementary energy acquisition such as aerobic anoxygenic photosynthesis (AAP), carbon monoxide (CO), and sulfur oxidation, but lacks plasmids, prophages, or complete GTAs (gene transfer agent) [6, 18, 19]. The other seven available isolates were also obtained from coastal waters [16, 17, 20], but genome sequences of these strains have not been published. Therefore, the genomic information on the RCA cluster is still scarce and hardly provides a comprehensive understanding of the genetic diversity and metabolic potential within the RCA cluster. Hence, we hypothesize that other members of the RCA cluster, dwelling exclusively in oceanic off shore regions, differ in their genomic features from the known isolates [2, 21].

For the last two decades, our understanding of the prokaryotic diversity and metabolic potential has greatly advanced by reconstructing genomes directly from environmental samples. Metagenome-assembled genomes (MAG) and single-cell amplified genomes (SAG) make it possible to gain insight into genomic and functional traits of uncultivated lineages, otherwise not accessible to genomic analyses [19, 22–26]. A nearly complete RCA genome from the Southern Ocean, co-assembled from three SAGs with identical 16S rRNA genes, was recently reported [21]. This genome, distinctly different from the genomes of the known RCA isolates, lacks many pathways for nitrogen (N) and phosphorus (P) uptake and metabolism and contains genes encoding proteorhodopsin (PR) and iron transporters. The latter genomic traits were proposed as a strategy of this RCA member to live in high-nutrient, low-chlorophyll regions [21]. We hypothesize that recruiting MAGs of the RCA cluster from different oceanic regions will be a valuable means to reveal the breadth of the functional potential of this important cluster and its phylogenomic diversity.

Therefore, we searched metagenomic and metatranscriptomic datasets of the Tara Ocean [27–29] and BioGEOTRACES expeditions [30], an Atlantic transect from 62°S to 47°N [31], cruises to the Arctic and Southern Ocean [32] and the North Sea, and collected MAGs affiliated to the RCA cluster from these metagenomes. This enabled us to carry out a comprehensive analysis of the genomic and functional traits of the RCA cluster, to assess its taxonomic diversity and the global biogeography of RCA sublineages.

## Materials and methods

### Metagenomic quality control, assembly and binning

We collected 127 and 7 RCA MAGs from the Ocean Microbiomics Database [29] and the North Sea (https://www.ncbi.nlm.nih.gov/bioproject/PRJNA365016), respectively, based on the genome taxonomy database (GTDB) (Supplementary Table S1). Besides, 20 MAGs were reconstructed from samples of an Atlantic Ocean transect from 62°S to 47°N (*n* = 22, Supplementary Table S1) [31]. Metagenomics data of this transect were quality controlled using Trimmomatic 0.36 [33] and sequencing reads assembled with metaSPAdes [34, 35]. Contigs ≥ 210 bp and with an average coverage > 2 were kept for metagenomic binning. MetaBAT2 (v2.12.1) [36] was used to reconstruct MAGs which were classified taxonomically using GTDB-Tk [37]. The completeness and contamination for assessing the quality of genomes were examined by both CheckM and Anvi’o [38, 39]. Only MAGs with a mean completeness ≥ 70%, a mean contamination ≤ 5%, and having scaffolds with an N50 ≥ 10 Kb were kept for further analysis. Five genomes of isolates were also included, i.e., genomic sequences of four strains (*Roseobacter* bacterium LE17 [20], *Rhodobacteraceae* bacterium IMCC1909, *Rhodobacteraceae* bacterium IMCC1923, and *Rhodobacteraceae* bacterium IMCC1933 [16]) were sequenced by a combined approach using 454 pyrosequencing and Illumina sequencing. The genomes were assembled using Newbler v2.8 [40] and SPAdes v2.5.1 [34]. Gap closure was partially done by PCR and Sanger sequencing of the products. The genome of the isolate *Planktomarina temperata* RCA23 was obtained from Integrated Microbial Genomes and Microbiomes database [41, 42]. Three previously reported SAGs were excluded for analyses due to low completeness (66.21%, 42.48%, and 17.24) [21]. In total, we obtained a set of 87 MAGs and genomes after quality control (Table 1, Supplementary Table S2).

**Table 1:** Species ID, number of MAG per species (in parenthesis), approved names of the species and genera of the RCA cluster, the location of the MAGs used for denomination, mean genome size ± standard deviation (SD), mean number of coding sequences (CDS) ± SD and mean G + C content ± SD of 13 RCA species

Species ID (*N* = number of MAGs/genomes)	Name	Genus	Origin	Mean raw genome Size (Mbp) ± SD	Mean estimated genome Size (Mbp) ± SD	Mean CDS ± SD	Mean GC Content (%) ± SD
Species *C1* (3)	*Cand* norwegica	*Planktomarina*	Arctic Ocean	1.91 ± 0.17	2.44 ± 0.14	1754.7 ± 170.6	48.56 ± 0.2
Species *C2* (1)	*Cand* australis		Southern Ocean, ANT28-4, station 241	2.32	3.12	2122.0	46.36
Species *C3* (14)	*forsetii*^a^		North Sea, Helgoland	2.31 ± 0.28	2.64 ± 0.22	2208.9 ± 294.7	52.00 ± 0.5
Species *C4* (8)	*arctica*^a^		Arctic Ocean	2.30 ± 0.13	2.85 ± 0.19	2144.3 ± 131.5	47.91 ± 0.1
Species *C5* (7)	*antarctica*^a^		Southern Ocean, ANT28-4, station 241	2.60 ± 0.85	3.12 ± 0.18	2426.6 ± 90.1	47.96 ± 0.1
Species *C6* (6)	*temperata*		North Sea, type strain	3.09 + 0.29	3.09 + 0.29	3040.5 ± 329.8	53.70 ± 0.9
Species C7 (2)	*Cand* atlantica		Atlantic SW	2.17 ± 0.65	2.49 ± 0.15	2104.5 ± 60.1	53.55 ± 0.0
Species *C8* (1)	*Cand* helgolandica		North Sea, Helgoland	2.18	2.92	2112	53.22
Species *B1* (2)	*Cand* australis	*Pseudoplanktomarina*^*a*^	Southern Ocean, ANT28-4, station 241	1.97 ± 0.52	2.36 ± 0.12	1844.5 ± 37.5	43.69 ± 0.1
Species *B2* (23)	*karensis*^a^		Arctic Ocean	2.17 ± 0.11	2.34 ± 0.11	2042.3 ± 50.0	43.72 ± 0.1
Species *B3* (9)	*bipolaris*^a^		Southern Ocean, ANT28-4, station 241	1.86 ± 0.14	2.26 ± 0.08	1707.6 ± 117.8	44.10 ± 0.1
Species *B4* (9)	*atlantica*^a^		Atlantic Ocean	2.20 ± 0.19	2.72 ± 0.12	2013.9 ± 180.5	42.22 ± 0.2
Species *A1* (2)	*Cand* aphotica	*Paraplanktomarina*	Arctic Ocean	1.93 ± 0.21	2.40 ± 0.11	1774.5 ± 195.9	45.85 ± 0.0

^a^The names were approved by the SeqCode

### Genome annotation and metabolic pathway prediction

Gene open reading frames and gene function were predicted with Prokka (default setting) [43]. Metabolic reconstruction of MAGs was performed by Anvi’o v.7.0 [39]: First, annotation of genes with the KEGG KOfam database was done by calling “anvi-run-kegg-kofams” [44] and the “anvi-estimate-metabolism” was then called for metabolic capabilities based on the KEGG MODULE database [45, 46]. A module with a completeness score ≥ 75% was considered as “present” in a genome.

### Phylogenetic analyses

#### Genome-based phylogeny of the RCA cluster

Overall, 87 RCA and two outgroup genomes (*Rhodobacterales* Bacterium HTCC2255 and *Rhodobacteraceae* bacterium HTCC2150) were included in the phylogenomic analysis. The phylogenetic tree was inferred from 120 bacterial marker genes [47]. The identification and alignment of marker genes and trimming of a concatenated alignment followed the GTDB-Tk workflow [37]. The concatenated alignment was applied to reconstruct a maximum likelihood (ML) phylogenetic tree using IQ-TREE [48] under the LG + R10 substitution model with 1000 ultrafast bootstraps. The tree was visualized using the Interactive Tree of Life view (iTOL) [49].

#### Phylogenetic tree construction of the PR and pufM genes

For construction of the PR phylogenetic tree, 591 PR-encoding amino acid sequences were downloaded from the Reference Sequence (RefSeq) database at NCBI (https://www.ncbi.nlm.nih.gov) [50]. After clustering at 90% amino acid sequence similarity using CD-HIT [51], 338 PR representative reference sequences were kept. Sequences of 68 PR genes extracted from the analyzed RCA genomes, along with references, were aligned with MAFFT and trimmed with trimAL “-gappyout” [52, 53]. IQ-Tree was used for a maximum likelihood phylogenetic tree with 1000 ultrafast bootstraps using the LG + F + R8 model [48]. A similar workflow was applied for the *pufM* gene phylogeny. This analysis included 43 sequences of the *pufM* gene, consisting of 34 references from the NCBI database and nine sequences of three *Planktomarina* species. An alignment of the *pufM* gene sequences was constructed with MAFFT and trimmed with trimAL [52, 53]. The phylogenetic tree was built using IQ-Tree using the LG + I + G4 model with a bootstrap of 1000 replicates [48].

#### Taxonomic classification according to the SeqCode nomenclature

Reference MAGs of the newly identified species which passed the quality control criteria of the SeqCode nomenclature, ≥ 90% completeness, ≤ 5% contamination [54], were submitted to the SeqCode nomenclature and confirmed as new validly described species.

### Global biogeography

To provide a global distribution pattern of the RCA cluster, we collected datasets including the Tara Ocean (370 metagenomic and 187 metatranscriptomic samples) [27, 28, 52], an Atlantic Ocean transect from 62°S to 47°N (22 metagenomic samples) [31], BioGEOTRACES expeditions (480 metagenomic samples) [30], and cruises to the Arctic and Southern Ocean (60 metagenomic samples) [32] and investigated the metagenomic operational taxonomic units (mOTUs) profiles of these samples [55]. Overall, we detected mOTUs corresponding to the RCA cluster in 282 samples (214 metagenomic, 68 metatranscriptomic) from the epipelagic zone (< 200 m), in 41 samples (36 metagenomic, 5 metatranscriptomic) from the mesopelagic zone (200–1000 m) and in 10 metagenomic samples from the bathypelagic zone (> 1000 m) (Supplementary Tables S3, S4).

The mOTUs based on housekeeping genes can identify microbial taxa at species-level resolution in metagenomes [55]. Therefore, we explored the distribution of the distinct RCA species in the global ocean based on mOTUs. Eleven mOTUs associated with members of the RCA cluster were collected in the mOTUs references database. Nine of the 11 mOTUs correspond to 10 of the 13 RCA species we newly identified as two species shared the same mOTUs. The remaining three species lack representatives in the mOTUs reference database.

## Results and discussion

### Diversity of the RCA cluster and genome characteristics

**Fig. 1:**
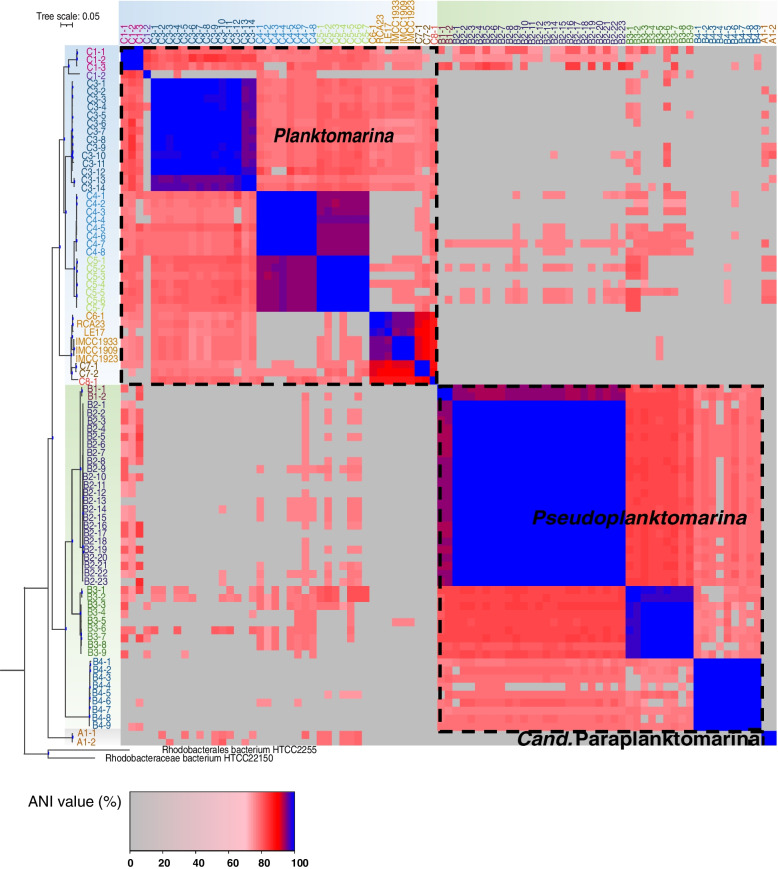
Phylogenetic tree and delineation of the three genera of the RCA cluster. Phylogenetic tree of the 82 MAGs and 5 genomes of isolates of the RCA cluster is based on 120 conserved genes and *Rhodobacterales* bacterium HTCC 2255 and *Rhodobacteraceae* bacterium HTCC 2150 were used as outgroups. Only bootstrap values ≥ 75 were shown with filled blue circles. The 13 species are shown in different colors. Heatmap of paired comparisons of Average Nucleotide Identity (ANI) among 87 RCA genomes/MAGs revealing three genera (ca. ANI > 70%), *Cand*. Paraplanktomarina, *Pseudoplanktomarina*, *Planktomarina*, and 13 species (A1, B1-B4, C1-C8) (ANI > 95%)

**Fig. 2:**
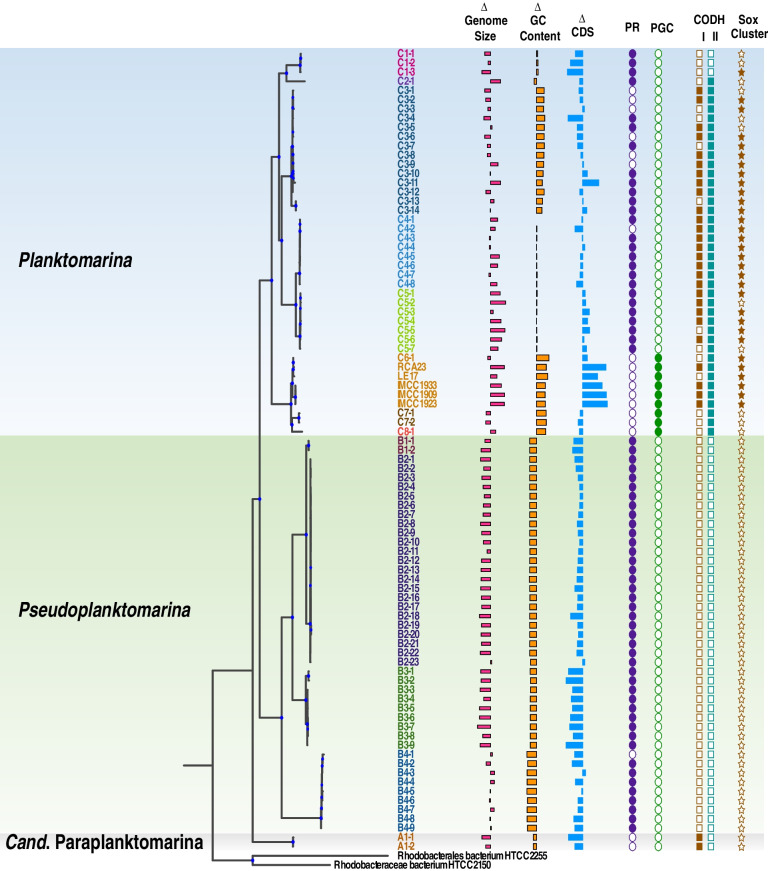
Genome characteristics of 87 RCA MAGs/genomes of the RCA cluster. Deviation of each genome/MAG from the overall mean of the genome size, G + C content and CDS and presence/absence of genes for complementary energy acquisition by proteorhodopsin (PR), aerobic anoxygenic photosynthesis (pufM), carbon monoxide dehydrogenase (CODH, i.e., Form I coxMSL and Form II coxSLM), and sulfur oxidation (sox cluster). Deviation from the mean is indicated by bar length and presence/absence of genes by filled or empty circles

The phylogenomic analysis yielded three major clades within the RCA cluster (Fig. 1) Genomes of the three clades were relatively distinct with appr. < 70% average nucleotide identity (ANI), resulting in the proposal of three genera, the known genus *Planktomarina*, and two new genera without representative pure cultures, named *Paraplanktomarina* and *Pseudoplanktomarina* (Fig. 2). *Pseudoplanktomarina* is an approved new genus by the SeqCode nomenclature (https://disc-genomics.uibk.ac.at/seqcode/page/seqcode), whereas *Paraplanktomarina* is a candidate genus*.* To explore whether the MAGs/genomes presented novel species, we clustered 82 MAGs plus 5 genomes of the isolates on the basis of 95% ANI, following previous studies [56, 57] and delineated 13 species-level genome clusters among the three RCA genera (designated A1, B1-B4, C1-C8; Fig. 2). ANI was estimated based on the whole-genomes comparisons using the FastANI [58]. In species *C3*, MAGs 13 and 14 formed a separate cluster with an ANI of 95.4–97.9% when compared to the other MAGs of this species. The three strains IMCC1909, IMCC1923, and IMCC1933, isolated from the Korean Yellow Sea, formed one cluster within the species *Planktomarina temperata*, sharing ANI values of 94.8–95.8% with strain LE17 and the type strain of this species.

*Cand.* Paraplanktomarina is the most deeply branching genus and represented by only one species with two MAGs. *Pseudoplanktomarina* encompasses four species with 43 MAGs, including species *B2* with the largest number of MAGs, and *Planktomarina* encompasses eight species with 43 MAGs/genomes. The five genomes of the isolates, including the type strain *P. temperata* RCA23, and one MAG formed a monophyletic group assigned to *Planktomarina C6* (Fig. 2). From nine RCA MAGs/genomes we obtained the 16S rRNA gene that has > 98% sequence similarity.

The genome size of the RCA species, corrected for contamination and completeness according to reference [59], ranges from 2.13 to 3.33 Mbp (Table 1). The genomes contain 1535 to 3310 coding DNA sequences (CDS) and the G + C content varies from 42.05 to 55.21% (Table 1). The genome size and G + C content of species *A1* of the most deeply branching genus, *Cand.* Paraplanktomarina, are 2.40 Mbp and 45.85%, respectively, and the number of CDS is 1774 (Table 1, Supplementary Table S2). Comparing genomic characteristics with dRep [60], and using the means of all MAGs/genomes of each species, the number of CDS of all except one *Planktomarina* (*C1*) and *Pseudoplanktomarina* species (*B3*) is higher than that of *Cand*. Paraplanktomarina. Numbers of CDS of the other *Planktomarina* species range between 2112 and 3014, whereas numbers of CDS of the other *Pseudoplanktomarina* species did not exceed 2042 (Table 1). This indicates that the number of CDS of *Planktomarina* is significantly larger (*p*-value < 0.05) than that of *Pseudoplanktomarin* (supplementary Fig. S1). The genome size of the *Pseudoplanktomarina* species *B1* to *B3* ranges between 2.26 and 2.36 Mbp and that of species *B4* is 2.72 Mbp. Hence, three of the four species of this genus have a reduced genome size relative to *Cand*. Paraplanktomarina (and the overall mean of 2.68 Mbp), despite an increase in CDS, and only species *B4* has a larger genome size (Table 1, Supplementary Table S2). The genome sizes of seven *Planktomarina* species are larger than that of *Cand*. Paraplanktomarina (and the overall mean) and only that of species *C1* was slightly smaller, features in line with the CDS data. The G + C content of all *Planktomarina* species is higher than that of *Cand*. Paraplanktomarina and ranges between 46.36 and 53.79% with the largest values in species *C6* and *C7* (Table 1). In contrast, the G + C content of all *Pseudoplanktomarina* species is lower than that of *Cand*. Paraplanktomarina and ranges from 42.22 to 44.10%. Interestingly, the co-assembled genome of the three SAGs retrieved from the Southern Ocean [21] affiliates most closely to species *Cand*. Paraplanktomarina *A1* and ANI indicates that it is a separate species of this genus (Supplementary Fig. S2). Its estimated genome size is 3.47 Mbp and its G + C content 45.3% (Supplementary Table S5).

These findings show substantial differences among the three RCA genera regarding genome size, CDS and G + C content. Most *Planktomarina* species have a larger genome and more CDS and all have a higher G + C content than the species of both other genera. These are typical evolutionary genomic features for prokaryotes dwelling under relaxed growth conditions and largely driven by mutation and horizontal gene transfer [61–64]. In contrast, the reduced genome size of three and the lower G + C content of all *Pseudoplanktomarina* species relative to *Cand*. Paraplanktomarina are typical features of streamlining and resource-driven evolution towards saving N under strong N limitation such as in oligotrophic open ocean ecosystems [65–68]. Biogeographic patterns reflect these genomic adaptations of the different species (see below).

As A1 of *Cand*. Paraplanktomarina is the most deeply branching species of the RCA cluster, distinct in quite a few genomic features from the species of both other genera, we focus our analysis of functional features predominantly on those in the genera *Pseudoplanktomarina* and *Planktomarina* differing from *Cand*. Paraplanktomarina *A1*. As both MAGs of *A1* are only to 77 and 84% complete, several genes in this species are likely missing due to genome incompleteness. General genomic features of the type strain *P. temperata* RCA23, affiliated to species *C6*, also in comparison to the entire *Roseobacter* group, have been published previously [6].

In none of the RCA MAGs or genomes, we detected complete GTAs, any prophage, or plasmid except for one incomplete prophage in IMCC1909 (Supplementary Table S6). Even though we are aware of the difficulties to detect these features in MAGs [69, 70], these findings are in line with previous reports of features of RCA genomes. These features were interpreted as an adaptation to oligotrophic growth conditions in pelagic marine ecosystems [6, 17], in line with genome characteristics of other streamlined pelagic marine prokaryotes including the SAR11 and SAR116 clades [71, 72].

### Complementary energy acquisition

#### Utilization of light energy

**Fig. 3:**
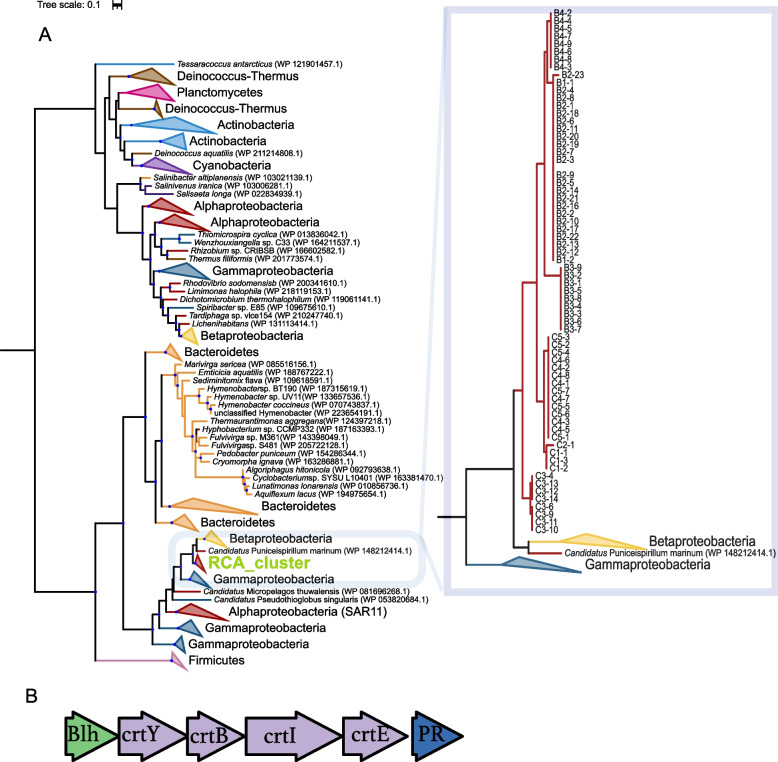
Phylogenetic tree of the PR gene and gene sequence of the PR operon detected in the RCA cluster. **A** Phylogenetic tree based on PR genes. **B** Gene sequence of the PR operon detected in the RCA cluster; *Blh*: 15,15′-β-carotene dioxygenase; *crtY*: lycopene cyclase; *crtB*: phytoene synthase; *crtI*: phytoene dehydrogenase; *crtE*: putative geranylgeranyl pyrophosphate (GGPP)

The *Roseobacter* group encompasses purely heterotrophic but quite a few subgroups including the RCA cluster carry out AAP whereas only one sublineage is known to encode the PR gene [6]. Therefore, it is of great interest to examine the MAGs of the RCA cluster for their genetic traits to acquire complementary light energy. *Cand*. Paraplanktomarina *A1* is likely to be purely heterotrophic as the two MAGs do not encode any gene for acquiring light energy (Fig. 2). In contrast, all *Pseudoplanktomarina* and five *Planktomarina* species and in total 68 MAGs encode the PR gene (Fig. 2). In a few genomes of these species, we did not find a PR-encoding gene but detected several genes involved in the retinal biosynthesis, presumably because their completeness is only 71 to 91%, making it likely that these MAGs miss this gene. It is surprising that nine of the twelve species in both genera are able to acquire light energy via PR. This mode of complementary energy acquisition by a proton pump is widespread in several lineages of marine pelagic bacteria, including the alphaproteobacterial SAR11 (*Pelagibacterales*) and SAR116 (*Puneispirillales*) clades, several lineages of *Gammaproteobacteria* and *Flavobacteriaceae* [73]. In the *Roseobacter* group, PR has only been reported to be encoded in the genome-streamlined NAC11-7 lineage, dwelling in pelagic systems [74], and from two SAGs affiliated to the RCA cluster retrieved from the Southern Ocean [21]. As we identified the latter as a species of the genus *Cand*. Paraplanktomarina (see above, Supplementary Fig. S2), it is likely that this most deeply branching genus or sublineages of it also acquire complementary energy by this light-driven proton pump. Hence, our findings indicate that this mode of complementary energy acquisition is widespread in the RCA cluster. As the most common ancestor of the RCA cluster, *Nereida ignava*, does not encode the PR gene (Supplementary Fig. S3, [75]), it is likely that members of the RCA cluster gained the PR gene by horizontal transfer from other prokaryotic lineages. Horizontal transfer of the PR gene is a quite common phenomenon between bacteria, not only of closely related taxa but also between families and even phyla [73]. To identify source candidates of this gene, we calculated a phylogenetic tree of bacterial PR genes (Fig. 3). This analysis showed that the 68 PR genes associated with the RCA cluster formed one clade, phylogenetically most closely related to members of *Betaproteobacteria* and *Cand.* Puniceispirillum marinum of the SAR116 clade (*Alphaproteobacteria*). Betaproteobacterial lineages are prevalent in freshwater habitats and have low abundances in the ocean [76, 77]. By contrast, the SAR116 clade is globally distributed in the oceans, accounting for up to 30% of prokaryotic communities [72]. Hence, the co-occurrence of the deeply branching RCA species, dwelling in nutrient depleted oceanic environments, with members of the SAR116 clade such as *Cand*. P. marinum, suggests a higher likelihood of gene transfer between these two phylogenetic lineages. Furthermore, the presence of mobile genetic elements near the PR gene in the RCA cluster (Supplementary Fig. S4) and the dissimilarity in GC content of the PR gene and its corresponding genome of the RCA cluster (Supplementary Table S7) further imply that the PR gene was transferred from a member of the SAR116 clade to members of the RCA cluster [78–80].

The PR gene becomes only functional when it is embedded in an operon including also genes encoding for the biosynthesis of different carotenoids and which can vary in their organization [73]. Hence, we analyzed the structure of the PR operon in the RCA MAGs and identified one type *Blh*-*crtY*-*crtB*-*crtI*-*crtE*-*PR* (Fig. 3). The analysis of the amino acid variants indicates that all PR genes affiliated with the RCA cluster are green-tuned [80], in line with the predominant occurrence of the RCA cluster in near surface waters [4].

All MAGs of *Planktomarina C6*, *C7* and *C8* encode the genes to synthesize bacteriochlorophyll *a* (BChl *a*) and the complete photosynthetic gene cluster (PGC) to carry out AAP (Fig. 2). *Cand*. Paraplanktomarina does not encode the PGC. Interestingly, the most closely related *Roseobacter* lineage of the RCA cluster, represented by *Nereida ignava*, and *Nereida* sp. MMG025, encodes the PGC as shown by its *pufM* marker genes (Supplementary Figs. S2, S4A [75]). However, it is encoded in a plasmid and not closely related to the PGC of the RCA cluster (Supplementary Fig. S5A, Supplementary Table S8). Hence, despite a PGC cluster being encoded in the most closely related lineage of the RCA cluster, it appears unlikely that the *Planktomarina* species *C6*, *C7*, and *C8* conserved it from a common ancestor. It is more likely that they acquired it by horizontal gene transfer from another *Roseobacter* lineage with a more closely related PGC. A phylogenetic analysis of the *pufM* genes shows that they form a monophyletic branch in the *Roseobacter* group with a subcluster of the *Planktomarina* species (Supplementary Fig. S5A). Two types of PGC organization were present: one in a genome and a MAG of *Planktomarina C6* and *C8*, isolated from the Korean Yellow Sea and North Sea, respectively; the other type in *Planktomarina C7* and in the other genomes of *Planktomarina C6* (Supplementary Fig. S5B). Both types have also been found in other species of the *Roseobacter* group. The AAP mode to acquire complementary energy is more cost-intense than the PR proton pump but conserves more energy than the latter [81]. The species carrying the PGC exhibit the highest G + C content (~ 53%) of all RCA species, suggesting that they are adapted to relatively relaxed resource conditions [67, 68] such as in coastal seas and the temperate and (sub)polar regions. These conditions are in line with distribution patterns of the RCA cluster reported previously, based on the detection of the 16S rRNA gene and genomic features of the isolate *P. temperata* RCA23 [2–4, 6, 11, 13].

The detection of genes encoding the PGC in MAGs of the RCA cluster was expected and is in line with previous reports and also with the fact that quite a few sublineages of the *Roseobacter* group encode AAP [6, 16]. The finding of PR in other species and in two of the three genera of the RCA cluster was unexpected as it was not detected in any RCA isolate and only reported in two SAGs (see above). To find both modes of complementary energy acquisition from light in one sublineage on the genus level of marine bacteria is unusual and to the best of our knowledge unprecedented. Our findings demonstrate that the RCA cluster subdivides into two fractions regarding acquisition of complementary light energy for adaptation to environmental conditions and resource limitation. In the sun-lit ocean, PR-based photoheterotrophic bacteria are more abundant than AAP bacteria in particular in oligotrophic regions [81]. It indicates that the majority of species of the RCA cluster are members of these photoheterotrophic prokaryotic communities dwelling predominantly in nutrient-poor oceanic environments. Surprisingly, only a small fraction of the RCA cluster belongs to the AAP bacterial communities of which the entire *Roseobacter* group can represent up to ~ 60% [8].

### Oxidation of carbon monoxide and reduced sulfur compounds

Other ways to conserve complementary energy in the *Roseobacter* group are oxidation of CO and reduced sulfur compounds including thiosulfate [8, 19, 82–84]. The *cox* gene cluster, encoding for the CO dehydrogenase (CODH), has two distinct types, form I and II [84, 85]. Form I contains genes in the order *coxMSL*, whereas form II in the order *coxSLM*. Only those *Roseobacter* organisms oxidize CO and transcribe *coxL* which encode both forms [82]. *Cand*. Paraplanktomarina *A1* encodes only CODH I (Fig. 2) and thus is predicted to be unable to oxidize CO. All *Pseudoplanktomarina* species and *Planktomarina C1* lack CODH I as well as form II, whereas most MAGs/genomes of the other *Planktomarina* species encode CODH I (Fig. 2). All MAGs/genomes of the *Planktomarina* species *C2* to *C8* encode CODH II. Hence, species *C3* to *C6* are predicted to oxidize CO. The fact that several MAGs of these species lack form II may be due to the incomplete genomes. *Planktomarina temperata* RCA23 has been shown to be capable of oxidizing CO. In cultures grown in the dark and supplemented with CO, cell numbers in the stationary phase remained significantly higher than in an unsupplemented control [86]. However, cell yield in the stationary phase was much smaller as compared to cultures grown at light relative to a dark control indicating that complementary energy acquisition by CO oxidation is much lower than by AAP. This suggests that other *Planktomarina* species with both forms may also take advantage of CO oxidation as complementary energy source, however, presumably only under extremely resource-limited conditions.

The *sox* cluster (*soxAXYZBCD*), encoding genes for acquiring energy via the oxidation of reduced sulfur compounds such as thiosulfate, is neither found in the *Cand*. Paraplanktomarina nor in *Pseudoplanktomarina* species and also not in *Planktomarina C2*, *C7*, and *C8*. Most MAGs/genomes of the other *Planktomarina* species, however, encode the *sox* cluster, which is also present in the closely related *Nereida ignava* [75].

In summary, the analyses of the different modes of complementary energy acquisition show a clear dichotomy in the speciation and diversification of the RCA cluster into the genera *Planktomarina* and *Pseudoplanktomarina*/*Cand*. Paraplanktomarina. *Planktomarina* has the highest G + C content, the largest genomes, five species encode the CODH I and II and *sox* genes, and three species harbor the PGC, whereas the other five species encode the PR gene. *Pseudoplanktomarina* does not encode CODH I and II nor *sox* genes but gained the PR gene. *Cand*. Paraplanktomarina does not encode CODH II but gained the PR gene in one sublineage [21].

### Utilization of organic carbon, nitrogen, phosphorous, sulfur and iron

**Fig. 4:**
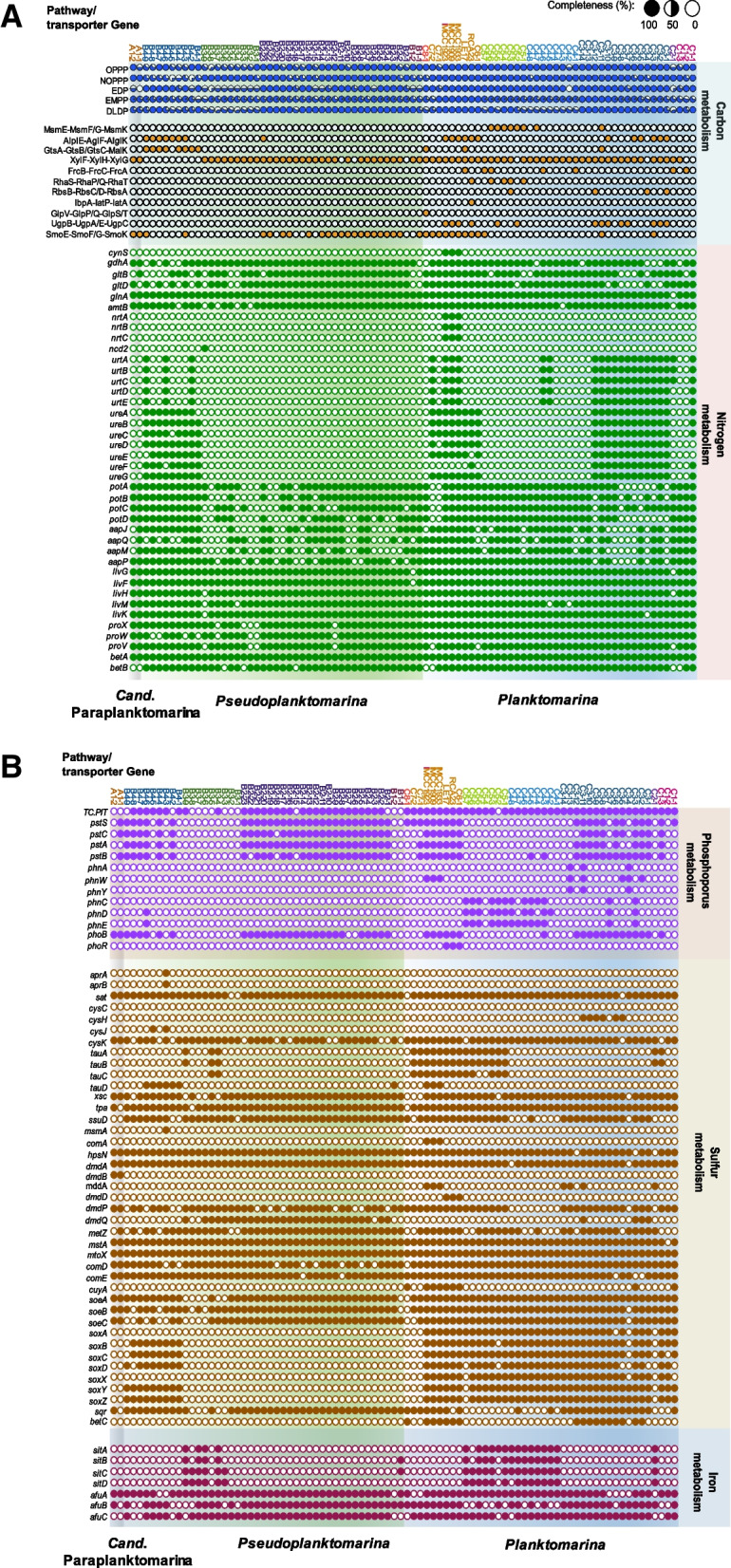
Overview of the genetically encoded metabolic potential of the 13 species and MAGs/genomes of the RCA cluster. **A** Carbon and nitrogen metabolisms. **B** Phosphorus, sulfur, and iron metabolism. For abbreviations of the listed genes, see Supplementary Table S9

For glycolysis, the Entner-Doudoroff (ED) and the pentose phosphate (PP) pathways are encoded in all species of the RCA cluster, whereas the Embden-Meyerhof-Parnas (EMP) pathway is incomplete and presumably not functional (Fig. 4A). This is consistent with findings for many members of the *Roseobacter* group and other marine bacteria including the SAR11 clade [71, 87]. Furthermore, the De Ley-Doudoroff (DLD) pathway for galactose catabolism is encoded in all RCA species but to different degrees of completeness. In species *B3*, *C2*, and *C4*, it is less complete than in the others leading to the speculation that the different species vary in their galactose metabolism.

RCA members harbor various ABC transporter systems for carbohydrate uptake, but *Paraplanktomarina A1* is most limited and only encodes a sorbitol/mannitol and a xylose transporter (Fig. 4A). Sorbitol and mannitol are osmoprotectants and transporters for both compounds are genetically encoded in many marine bacteria [88]. These transporters are encoded in MAGs/genomes of several *Pseudoplanktomarina* and *Planktomarina* species including species *C6* to *C8* and *B2* (*smoEFGK*), whereas species *C1*, *C2*, *C4*, and *B3* do not encode them. They must rely on other osmoprotectants, such as choline and glycine-betaine (see below). The gene of the transporter for xylose (*xylFHG*) is encoded in the MAGs/genomes of all RCA species except *B4* (Fig. 4A). Xylose is a major constituent of phytoplankton-derived polysaccharides [89, 90] and a potentially important carbon source of many marine bacteria. Even though the *Roseobacter* group is deficient in polysaccharide hydrolysis [91] and relies on other polysaccharide-degrading bacteria such as *Flavobacteriia* and different lineages of *Gammaproteobacteria* [90], xylose can be utilized by various *Roseobacter* lineages [91] and is predicted to be utilized by all species of the RCA cluster except *B4*. This species, however, encodes a transporter for alpha-glucosides and glucose/mannose. Alpha-glucoside transporters (*aglEFK*) and glucose/mannose transporters (*gtsABC*, *malK*) were only encoded in MAGs/genomes of two other species, *C3* and *C6* (Fig. 4A). In addition, several other transporters for carbohydrates are encoded in a few MAGs of other RCA species but are not of general significance (Fig. 4A). These data indicate that the utilization of different monosaccharides is rather limited among the members of the RCA cluster. Several *Pseudoplanktomarina* and *Planktomarina* species encode transporters for one or a few monosaccharides but without a phylogenetic consistency. It appears that the transporters reflect an adaptation to species-specific environmental or biotic conditions.

Acquisition and utilization of organic N compounds by *Cand*. Paraplanktomarina *A1* and most other species, based on genomic predictions, include the ammonium transporter (*amtB*), glutamate synthase (*gltB*, *gltD*), glutamine synthetase (*glnA*), and glutamate dehydrogenase (*gdhA*) (Fig. 4A). Furthermore, these species encode transporters for different amino acids (*aapJQMP* and *livGFHMK*) and polyamines (*potABCD*), but the latter are missing in a few MAGs of several species. Most species also encode genes for the metabolism of choline to glycine-betaine (*betAB*) and the glycine betaine transporter (*proXWV*) (Fig. 4A), presumably the major osmoprotectant of these organisms. Species *C3*, *C6*, and *C8* additionally encode the choline sulfatase (*betC*), thus further widening the use of osmoprotectants of these species with the highest G + C content, presumably dwelling under relatively relaxed resource conditions and also at lower salinities [3]. Species *B4*, *C1*, *C3*, *C6*, and *C7* encode also a urease (*ureABCDEFG*) and *C3* and one or several MAGs of species *B4*, *C1*, *C2*, *C4*, *C6*, and *C7* also a urea transporter (*urtABCDE*), indicating that most of these species are able to utilize urea as an N source (Fig. 4A). Urea has been shown to be an important source of organic N for marine pelagic prokaryotes [92]. Our findings show that this is also the case for the majority of RCA species. Interestingly, the isolates of *Planktomarina C6* from the Korean Yellow Sea in addition encode a cyanate lyase (*cynS*) and nitrate transporters (*nrtABC*), indicating that these coastal species further broadened their potential for acquiring N compounds and thus their niche space.

Regarding utilization of phosphorous (P), it is interesting that *Cand*. Paraplanktomarina *A1* encodes only genes of the high-affinity phosphate transporter (*ptsBCS*), detected; however, only in one of both MAGs of this species, and the phosphate regulon gene *phoB* (Fig. 4B). Hence, this species is predicted to be rather limited in its P-acquisition traits, whereas the species of the other genera exhibit additional transporters for inorganic and organic P. The low affinity P-transporter (TC.PIT), widely distributed in *Bacteria* [93], is encoded in all other species, except *B1*, even though detected only in two MAGs of *Pseudoplanktomarina B3* (Fig. 4B). Genes encoding the high affinity P-transporter (*ptsABCS*) were detected in most species but not in species *B1*, *B3 C2*, *C4*, and *C5*. The phosphate regulon (*phoBR*) is also rather widely genetically encoded but not present in species *B1*, *B3*, *C2*, *C5*, *C7*, and *C8* and only in one MAG of species *C4* (Fig. 4B). In contrast, genes encoding the carbon-P lyase (*phnABCDEWY*) were only detected in MAGs/genomes of species *C3*-*C6* (Fig. 4B). It was puzzling, though, that we did not detect any P-acquisition genes in *Pseudoplanktomarina B3* except in two MAGs. We assume that due to the completeness of the genomes of this species of only 72 to 89% (Supplementary Table S2) these genes were not captured. Our findings indicate that species of the RCA cluster are able to acquire P from the two major pools of dissolved organic P in the ocean, organophosphoesters and organophosphonates [94], but there is a clear separation of species exploring either pool except *Planktomarina C3* to *C5*, which can access both pools. *Planktomarina* species are more versatile in P-acquisition than species of the other two genera.

The *Roseobacter* group7 is known to be one of the most active players in marine environments in the cycling of organic sulfur compounds such as dimethylsulfonium propionate (DMSP) [19] and dihydroxypropane sulfonate (DHPS) [95]. Therefore, it was not surprising to find the key genes of the DMSP-demethylation and cleavage pathways (*dmdA*, *dddP*) and of DHPS-degradation and subsequent sulfonate oxidation (*hpsN*, *comDE*, *soeABC*) in many species of all three genera (Fig. 4B) [96, 97]. This indicates that they are involved in the breakdown of these important organic sulfur compounds. The methanethiol (MeSH) oxidase (*mto*) is encoded as well, indicating that these species are able to oxidize MeSH [98]. They further encode both enzymes for biosynthesis of cysteine from serine, cysteine synthase (*cysK*), and L-serine O-acetyltransferase (*sat*). In addition, the 3-mercaptopyruvate sulfurtransferase (*mstA*) is genetically encoded and involved in other metabolic processes of organic sulfur compounds. Taurine is an important C-, N-, and potentially S-source of marine bacterioplankton [99]. The proteins encoding taurine metabolism (*tauD*, *xsc*, *tpa*) [100] exist in almost all members of the RCA cluster, though the binding protein for taurine import (*tauABC*) is only encoded in MAGs/genomes of species *C1*, *C2*, *C5*, *C6*, and *B3* (Fig. 4B). Hence, a limited number of *Planktomarina* species and one *Pseudoplanktomarina* species are predicted to be involved in the cycling of this important compound. Furthermore, a few MAGs of *Planktomarina C3* contain the gene *cysH*, encoding a phosphoadenosine phosphosulfate reductase, and the genomes of *Planktomarina C6 cuyA*, encoding the desulfonation of cysteate to pyruvate and ammonium. Hence, several species of the RCA cluster have an even larger metabolic potential of metabolizing reduced and oxidized organic sulfur compounds.

All species of the RCA cluster encode genes for ferric iron transporters (*afuABC*), while genes encoding importers for ferrous iron (*sitABCD*) are missing in most species (Fig. 4B). Only *Planktomarina C2*, *C4*, and *C5* and several MAGs of *Pseudoplanktomarina B3* encode proteins for the import of ferrous iron (Fig. 4B), which suggests that they gained this trait by horizontal gene transfer.

### Global biogeography of RCA species

All RCA MAGs were obtained from the epipelagic zone of either temperate or (sub)polar regions, except two MAGs of *Pseudoplanktomarina B2* which were collected from the mesopelagic zone (Supplementary Table S2). Fifty-eight MAGs were collected from polar regions, of which 80% (47 MAGs) originated in the Arctic Ocean. Genomes/MAGs of species *C6* and *B4* came exclusively from temperate regions (Supplementary Table S2).

**Fig. 5:**
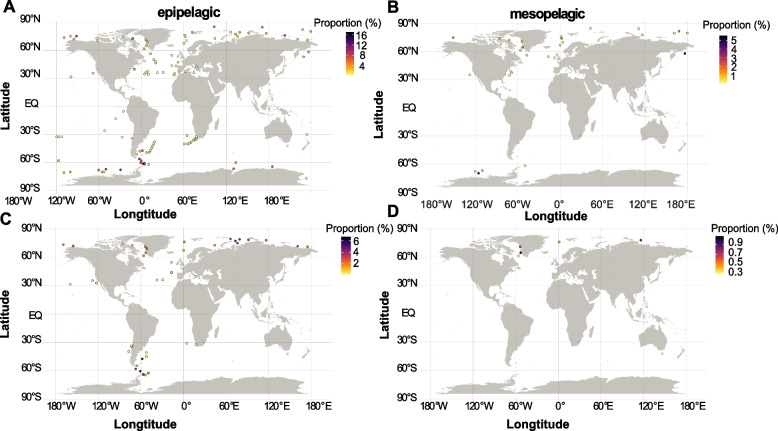
Global biogeography of the RCA cluster. **A**, **B** Relative abundance of the RCA cluster (% of total metagenomic reads mapping to mOTUs marker genes) in the epipelagic (0–200 m) and mesopelagic zones (200–1000 m). **C**, **D** Relative abundance of the RCA cluster (% of total metatranscriptomic reads mapping to mOTUs marker genes) in the epipelagic (0–200 m) and mesopelagic zones (200–1000 m). The dots in different colors indicate the percent of the RCA cluster. Note the different scalings of the colored scale on the panels

Based on taxonomic profiling of metagenomic reads, the relative abundance of the RCA cluster accounted for up to 16.9% and 5.3% of the total bacterial communities in the epipelagic and mesopelagic layers, respectively, with the highest percentages in the Southern Ocean (Fig. 5A, B, Supplementary Table S10). These proportions are in line with previous findings based on metagenomics and 16S rRNA marker gene studies [2–4, 6, 10]. Even at depths of more than 1000 m in the Southern Ocean, members of the RCA cluster account for up to 5% of the total bacterial community (Supplementary Table S10). Based on metatranscriptomic reads, the RCA cluster exhibits similar distribution patterns as on the basis of metagenomic reads, even though with fewer samples, indicating that this cluster is an active player of the resident prokaryotic communities (Fig. 5C, D, Supplementary Table S11).

**Fig. 6:**
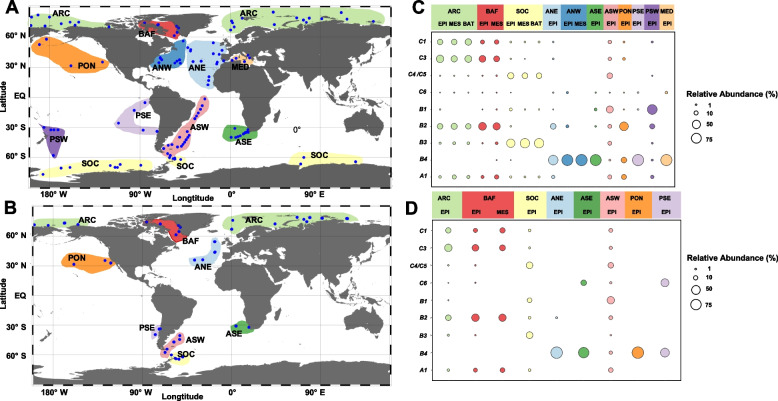
Oceanic regions and global distribution patterns of 10 species of the RCA cluster. **A**, **B** Distribution patterns of 10 RCA species in metagenomic and metatranscriptomic data in 11 geographic oceanic regions: Arctic Ocean (ARC), Baffin Bay (BAF), Southern Ocean (SOC), Atlantic north-east (ANE), north-west (ANW), south-east (ASE), and south-west (ASW), Pacific north (PNO); south-east (PSE) and south-west (PSW), Mediterranean Sea (MED). **C**, **D** Relative abundances (% of total RCA reads mapping to mOTUs marker genes) of the different species in the pooled metagenomic and metatranscriptomic samples of the different oceanic regions. Circle size refers to relative abundances of 1–75%

Three identified RCA species (*C2*, *C7*, *C8*) in the present study lack representatives in the mOTUs reference database, indicating that the proportion of the RCA cluster is underestimated. The global distribution and relative abundance of the other ten RCA species, however, could be assessed with mOTUs. Based on metagenomic and metatranscriptomic data, we identified eleven geographic regions of the Atlantic, Pacific, Arctic, and Southern Ocean and the Mediterranean Sea for the distribution patterns of these species (Fig. 6A, B). Most distinguished was *Pseudoplanktomarina B4*, which was abundant in temperate regions of the Atlantic and Pacific Ocean and the Mediterranean Sea and also detected at low relative abundance in Baffin Bay and the Southern Ocean but not at all in the Arctic Ocean (Fig. 6C, D, Supplementary Fig. S6). In the Mediterranean Sea, the northwest Atlantic and the southeast Pacific B4 was the only RCA species detected at most sampling stations (Supplementary Fig. S6, Supplementary Table S3). The high abundance of *Pseudoplanktomarina B4* in temperate regions, accounting for more than 50% and up to > 90% of the total RCA cluster, corresponds to the MAGs of this species recovered from the temperate Atlantic, indicating that this RCA species inhabits temperate oceanic regions. The abundance of the RCA cluster in the Atlantic north-west, Pacific south-east, and Mediterranean Seas is generally lower than in the temperate and (sub)polar regions (Fig. 5) [2, 4].

Species *C1*, *C3* to *C4/5*, *B3*, and *A1* were restricted to latitudes of > 40° in both hemispheres (Fig. 6C, D, Supplementary Fig. S6). Only *B1*, *B2*, and *C6* occurred also at lower latitudes, co-occurring with *Pseudoplanktomarina B4* in the south Atlantic but at lower abundances (Supplementary Fig. S6). *Pseudoplanktomarina B1* and *B4* were the only species detected in latitudes < 40° of the Pacific Ocean. As the Pacific Ocean was not well represented in our database, we are reluctant to conclude that the other species are really absent from the Pacific. All species except *B4* exhibited a bipolar distribution (Fig. 6C, D, Supplementary Fig. S6). However, species *B2*, *C1*, and *C3* exhibited relatively higher abundances in the Arctic Ocean, whereas species *B3* and *C4/5* were relatively more abundant in the Southern Ocean (Fig. 6C, D). Species *Paraplanktomarina A1* exhibited rather similar abundances in both polar oceans. In every geographic region, at least two species co-occurred, but in Baffin Bay, the Southern Ocean and south-west Atlantic even ten species. Biogeographic distribution patterns based on metatranscriptomic data matched those based on metagenomic data, but fewer stations were available for this analysis (Fig. 6B, D, Supplementary Tables S10 and 11). These data specify that RCA species are active players of the resident prokaryotic communities.

Bipolar distribution patterns of prokaryotic genera such as *Polaribacter*, *Octadecabacter*, subcluster Ia.1 of the SAR11 clade and the RCA cluster have been reported previously [2, 8, 101–103]. Our MAG-based approach reveals that even several species of the RCA cluster co-occur in both polar oceans despite the huge geographic distance. It has to be kept in mind, though, that this species definition is based on an ANI of > 95% [47] and that it relates to a MAG/genomes-species, not to a species definition in the classical microbiological sense [104]. Several of these species exhibit some within-species genomic variability (Fig. 2) which suggests that they underwent subspecies diversification. This subspecies adaptation to the environmental and biotic conditions and diversification was expected considering the well-separated water masses in both polar oceans. The SAR11 subcluster Ia.1 exhibits also some diversification, suggesting within-cluster adaptation to specific environmental conditions [103]. On the other hand, we detected several co-occurring RCA species at the same location in different geographic regions. However, this is not uncommon and known for other marine and freshwater prokaryotes as they may occupy different co-existing ecological niches which exhibit spatio-temporal dynamics in the course of phytoplankton blooms and/or seasonally. It 7has been shown by SAG analyses that different species and ecotypes of *Prochlorococcus* and the SAR11 clade co-exist [105–107].

### Proposal of species names

Considering the origin of the samples from which the MAGs originated and some biogeographic and functional features, we name the twelve new RCA species as indicated in Table 1. Six of the twelve species are validated by the SeqCode recently introduced to identify prokaryotes on the basis of a sequenced genome and other requirements [54]. Details and vouchers of the validly approved species are provided in the Supplementary text S1.

## Concluding remarks

Genome expansion and reduction were shown to be important events during the evolution of the *Roseobacter* group [108]. In the most ancestral lineages, gene loss dominated and led to reduced genome sizes, whereas in other more advanced lineages, gain of gene families dominated. However, gene loss and reduction of genome size persisted in several sublineages and resulted in a cluster of *Roseobacter* organisms, the Pelagic *Roseobacter* Cluster including the RCA cluster, with a gene content distinct from other lineages with larger genomes and occurring in pelagic marine systems [109]. Our detailed analysis of the 82 MAGs and genomes of five isolates of in total 13 species of the RCA cluster reveals that diversification, genome reduction, and expansion occurred also in this globally important pelagic lineage. In comparison to the most deeply branching *Paraplanktomarina A1*, a few *Pseudoplanktomarina* species further reduced the genome size and G + C content, well-known adaptations to resource-limited pelagic environments [61]. The number of CDS increased and genetic adaptations included several features, indicated, e.g., by the gain of the PR gene. In contrast, all species except one of the genus *Planktomarina* exhibited larger genomes, a higher G + C content and more CDS than both other genera, and more and different modes of complementary energy acquisition. This suggests the genera *Pseudoplanktonmarina* and *Planktomarina* likely evolved from *Cand*. Paraplanktomarina, but more in-depth analyses are necessary to support this hypothesis. The global biogeographic distribution patterns of the different RCA species reflect their genomic features to a great extent. Such studies are important for a more refined and better understanding of the speciation, environmental adaptation, and successful performance of relevant prokaryotic players in global elemental cycles.

### Supplementary Information

**Additional file 1:** **Supplementary text S1. Table S1.** Basic information of 154 MAGs of the RCA cluster. **Table S2.** Basic sequencing and genomic information of 87 analysed RCA MAGs/genomes (82 MAGs and 5 genomes from isolates) and locations where the metagenomic samples and isolates were collected. **Table S3.** Metadata for metagenomic data used to estimate the proportion of the RCA cluster and 10 of the 13 RCA species. **Table S4.** Metadata for metatranscriptomic data used to estimate the proportion of the RCA cluster and 10 of the 13 RCA species. **Table S5.** Genome data of Southern Ocean SAG [21]. **Table S6.** Annotation of genes encoded on PGC-containing extrachromosomal replicons of two Nereidia strains. **Table S6.** The incomplete prophage in the Rhodobacteraceae bacterium IMCC1909. **Table S7.** The GC content of genomes and PR genes in the RCA cluster. **Table S8.** Annotation of genes encoded on PGC-containing extrachromosomal replicons of two Nereidia strains. **Figure S1.** The variation of the genome size, GC content and CDS number in Planktomarina and Pseudoplanktomarina species. Values at the top indicate significant p-values. The significant difference was tested using the Wilcoxon test using the R package "rstatix" v0.7.2. **Figure S2.** Phylogenetic tree of southern Ocean SAG [21] and the RCA cluster. **Figure S3.** Phylogenetic tree of the RCA cluster with two Nereida strains as most closely related non-RCA species. **Figure S4.** Organization and structure of the PR operon present in genomes of RCA species. Blh: 15,15′-β-carotene dioxygenase; crt: carotenoid biosynthesis genes. **Figure S5.** Phylogenetic tree of the pufM gene and structure and arrangements of PGC operons detected in the RCA cluster. A: Phylogenetic tree based on 43 pufM genes. B: structure and arrangements of PGC operons detected in genomes of Planktomarina C6, C7 and C8. The tree was constructed using IQ-TREE under the LG+R10 substitution model with 1000 ultrafast bootstraps. Only bootstrap values ≥75 were shown with filled blue circles. **Figure S6.** Geographic distribution and relative abundance of ten of the thirteen RCA species (C1, C3-C6, B1-B4, A1) in different layers based on metagenomic and metatranscriptomic data: epipelagic (EPI, 0-200 m), mesopelagic (MES, 200-1000 m) and bathypelagic (BAT >1000 m). The dots in different colors indicated the relative abundance of the species as percent of the RCA cluster. Figure S6A - Metagenomics species A1, B1, B2. Figure S6B – Metagenomics species B3, B4/5. Figure S6C – Metagenomics species C1, C3, C4/C5, C6. Figure S6D – Metatranscriptomics species A1, B1, B2, B3, B4. Figure S6E – Metatranscriptomics species C1, C3, C4/5, C6.

## Data Availability

The genomes of strains *Rhodobacteraceae* bacterium IMCC1909, *Rhodobacteraceae* bacterium IMCC1923, *Rhodobacteraceae* bacterium IMCC1933, and *Roseobacter* bacterium LE17 have been deposited at NCBI under the following accession numbers: SUB12126367 PRJNA887231 SAMN31157516 JAPDHM000000000 (IMCC1909), SUB12126368 PRJNA887233 SAMN31157519 JAPDHN000000000 (IMCC1923), SUB12126370 PRJNA887234 SAMN31157520 JAPDHO000000000 (IMCC1933), and SUB12126372 PRJNA887235 SAMN31157521 JAPDHP000000000 (LE17). Genomes of 154 RCA MAGs that we collected are available on figshare (https://doi.org/10.6084/m9.figshare.21931311.v1). The results of gene function predicted with Prokka for the 87 RCA MAGs and genomes in this study are also deposited at figshare (https://doi.org/10.6084/m9.figshare.21931707.v1). Genomes submitted to SeqCode are available at the European Nuncleotide Archive (ENA) under project no. PRJEB62088 and PRJNA934655 and at the SeqCode registry under the following URLs: https://seqco.de/i:24046, https://seqco.de/i:24058, https://seqco.de/i:24063, https://seqco.de/i:24060, https://seqco.de/i:24064, https://seqco.de/i:24065. Sequences of *PR* and *pufM* genes for the phylogenetic tree are available on figshare (https://doi.org/10.6084/m9.figshare.21932034.v1; https://doi.org/10.6084/m9.figshare.21932100.v2). Samples for studying the global distribution of the RCA cluster are from published datasets. 370 metagenomes and 187 metatranscriptomes have been deposited in the NCBI database, and the information of these samples is available at https://doi.org/10.5281/zenodo.3473199. Metagenomics from the Atlantic Ocean and Polar regions are deposited in the European Nucleotide Archive (INSDC accession no. PRJEB34453) and NCBI database (BioProject accession no. PRJNA588686), respectively. Sequence data of GEOTRACES cruises are available in NCBI (BioProject accession no. PRJNA385854 and PRJNA385855).
