# Supplementary material for: Metagenome-assembled genomes reveal greatly expanded taxonomic and functional diversification of the abundant marine Roseobacter RCA cluster
Source: Microbiome. 2023 Nov 25;11:265. doi: 10.1186/s40168-023-01644-5 (PMC10675870; doi:10.1186/s40168-023-01644-5)
Supplement: Supplementary file 2 — Additional file 1: Supplementary text S1. Table S1. Basic information of 154 MAGs of the RCA cluster. Table S2. Basic sequencing and genomic information of 87 analysed RCA MAGs/genomes (82 MAGs and 5 genomes from isolates) and locations where the metagenomic samples and isolates were collected. Table S3. Metadata for metagenomic data used to estimate the proportion of the RCA cluster and 10 of the 13 RCA species. Table S4. Metadata for metatranscriptomic data used to estimate the proportion of the RCA cluster and 10 of the 13 RCA species. Table S5. Genome data of Southern Ocean SAG [21]. Table S6. Annotation of genes encoded on PGC-containing extrachromosomal replicons of two Nereidia strains. Table S6. The incomplete prophage in the Rhodobacteraceae bacterium IMCC1909. Table S7. The GC content of genomes and PR genes in the RCA cluster. Table S8. Annotation of genes encoded on PGC-containing extrachromosomal replicons of two Nereidia strains. Figure S1. The variation of the genome size, GC content and CDS number in Planktomarina and Pseudoplanktomarina species. Values at the top indicate significant p-values. The significant difference was tested using the Wilcoxon test using the R package "rstatix" v0.7.2. Figure S2. Phylogenetic tree of southern Ocean SAG [21] and the RCA cluster. Figure S3. Phylogenetic tree of the RCA cluster with two Nereida strains as most closely related non-RCA species. Figure S4. Organization and structure of the PR operon present in genomes of RCA species. Blh: 15,15′-β-carotene dioxygenase; crt: carotenoid biosynthesis genes. Figure S5. Phylogenetic tree of the pufM gene and structure and arrangements of PGC operons detected in the RCA cluster. A: Phylogenetic tree based on 43 pufM genes. B: structure and arrangements of PGC operons detected in genomes of Planktomarina C6, C7 and C8. The tree was constructed using IQ-TREE under the LG+R10 substitution model with 1000 ultrafast bootstraps. Only bootstrap values ≥75 were shown with filled [file 40168_2023_1644_MOESM1_ESM.zip › 40168_2023_1644_MOESM1_ESM.docx]

To be resubmitted to The Microbiome

Metagenome assembled genomes greatly expand functional genomics and species diversification of the pelagic *Roseobacter* RCA cluster

Yanting Liu^1,2,6*^, Thorsten Brinkhoff^1*^, Martine Berger^1^, Anja Poehlein^3^, Sonja Voget^3^, Lucas Paoli^4^, Shinichi Sunagawa^4^, Rudolf Amann^2^, Meinhard Simon^1, 5*^

^1^ Institute for Chemistry and Biology of the Marine Environment, University of Oldenburg, Carl von Ossietzky Str. 9-11, D-26129, Oldenburg, Germany

^2^ Max Planck Institute for Marine Microbiology, Bremen, Germany

^3^ Department of Genomic and Applied Microbiology & Göttingen Genomics Laboratory, Georg-August University Göttingen, Grisebachstr. 8, D-37077 Göttingen, Germany

^4^ Department of Biology, Institute of Microbiology and Swiss Institute of Bioinformatics, ETH Zürich, Zürich, Switzerland

^5^ Helmholtz Institute for Functional Marine Biodiversity at the University of Oldenburg (HIFMB), Ammerländer Heerstr. 231, D-26129 Oldenburg, Germany

^6^ State Key Laboratory for Marine Environmental Science, Institute of Marine Microbes and Ecospheres, Xiamen University, Xiamen, People’s Republic of China

*Authors for correspondence:

Yanting Liu: [yanting.liu@uni-oldenburg.de](mailto:yanting.liu@uni-oldenburg.de); [yantingl0702@xmu,edu.cn](mailto:yantingl0702@xmu,edu.cn)

Thorsten Brinkhoff: t.brinkhoff@icbm.de

Meinhard Simon: [m.simon@icbm.de](mailto:m.simon@icbm.de)

**Supplementary Text S1, Tables S1 to S11 (see extra Excel file) and Figures S1 to S6**

**Supplementary text S1**

**Description of *Planktomarina forsetii* sp. nov. (C3)**

for.set’i.i; N.L. gen. masc. n. *forsetii,* of Forseti, a god in Frisian mythology that lived on the North Sea island Helgoland from where the genome was recovered (North Sea close to Helgoland).

The type material, NSEA_SAMEA5407188_METAG_20160512B7G10 (MAG C3-11), is a metagenome-assembled genome derived from a water sample (Biosample: SAMEA5407188). The assembly is of high quality with a mean completeness of 96.74% and 0.10% contamination and contains 35 tRNA. The genome size is 3.12 Mbp (raw: 3.02 Mbp) with a GC content of 0.51.

**Description of *Planktomarina arctica* sp. nov. (C4)**

arc’ti.ca, L. fem. adj. *arctica*, northern, from the Arctic, corresponding to the origin from where the genome was recovered (Arctic ocean).

The type material, TARA_SAMEA4397426_METAG_DDEDKPON (MAG C4-4), is a metagenome-assembled genome from a sample taken at the epipelagic zone in the Arctic Ocean in 2013 (Biosample accession: SAMEA4397426). The completeness and contamination of the assembly are 93.42% and 0.41%, respectively. Genome assembly contains 21 tRNA genes. The genome size is 2.64 (raw: 2.47 Mbp) with a GC content of 0.48.

**Description of *Planktomarina antarctica* sp. nov. (C5)**

an.tarc’ti.ca. L. fem. adj. *antarctica*, of the Antarctic, corresponding to the origin from where the genome was recovered (Antarctic Ocean).

The type material, ANT28_SAMPLE241_METAG_CPIDHMEF (MAG C5-3), is a metagenome-assembled genome from a sample taken at the epipelagic zone in the Southern Ocean in 2012 (Biosample accession: SAMEA5958381). The assembly genome is of high quality with 94.16% completeness and 1.77 % contamination and contains 27 tRNA genes. The genome size is 2.80 Mbp (raw: 2.68 Mbp) with a GC content of 0.48.

**Description of *Pseudoplanktomarina* gen. nov.**

Pseu.do.plank.to.ma.ri'na. Gr. neut. adj. *pseudes*, false; N.L. fem. n. *Planktomarina*, a marine bacterial genus; N.L. fem. n. Pseudoplanktomarina, false *Planktomarina*

Members of *Candidatus* Pseudoplanktomarina genus are aerobic heterotrophic bacteria with an average genome size 2.41 Mbp (raw: 2.17 Mbp) and a GC content of 0.43. Currently, there are three distinct species exhibiting different distribution patterns across the global ocean. All species in this group contain a proteorhodopsin gene but lack genes of the CODH I and II cluster, two species (B2, B3) lack genes for sox clusters. Entner-Doudoroff and pentose phosphate pathways are two major pathways for glycolysis catabolism in *Candidatus* Pseudoplanktomarina. Additionally, genes involved in DMSP and DHPS degradation are also present in this genus. Two species within *Candidatus* Pseudoplanktomarina (B2, B3) have limited potential for urea uptake and metabolism.

**Description of *Pseudoplanktomarina karensis* sp. nov. (B2)**

kar.en’sis. N.L. masc./fem. adj. *kara*, pertaining to the Kara Sea, corresponding to the origin from where the genome was recovered (Kara Sea).

The type material, TARA_SAMEA4397239_METAG_COHLBNIA (MAG B2-20), is a metagenome-assembled genome from a sample taken at the epipelagic zone in the Kara Sea, Arctic Ocean (Biosample accession: SAMEA4397239). The assembly genome is of high quality with 95.60 % completeness with 0.02 % contamination and the genome contains 29 tRNA genes. The genome size is 2.28 (raw: 2.18) with a GC content of 0.44.

**Description of *Pseudoplanktomarina bipolaris* sp. nov. (B3)**

bi.po.la’ris. L. adv. *bis*, twice; M.L. masc./fem. adj. *polaris*, polar; bipolaris**,** pertaining to both poles, corresponding to the origin from where the reference and closely related genomes were recovered (North and South Pole).

The type material, ANT28_SAMPLE241_METAG_CPHJOAPH (MAG B3-5), is a metagenome-assembled genome from a sample taken at the epipelagic zone in the Southern Ocean in 2012 (Biosample accession: SAMEA5958381). The completeness and contamination of the assembly are 89.08 % and 0.20 %, respectively. Genome assembly contains a partial 5S rRNA gene (79 bp) along with 22 tRNA genes. The genome size is 2.21 Mbp with a GC content of 0.44.

**Description of *Pseudoplanktomarina atlantica* sp. nov. (B4)**

at.lan’ti.ca. L. fem. adj. *atlantica*, of or pertaining to the Atlantic Ocean, corresponding to the origin from where the reference genome was recovered (Atlantic Ocean).

The type material, BGEO_SAMN07136937_METAG_ALPGGBFO (MAG B4-3), is a metagenome-assembled genome from a sample taken at the epipelagic zone in the Atlantic Ocean 2012 (Biosample accession: SAMN07136937). The completeness and contamination of the assembly are 89.94 % and 1.02 %, respectively. The genome assembly contained a complete 5S rRNA gene along with 26 tRNA genes. The genome size is 2.86 Mbp with a GC content of 0.42.

**For Tables S1 to S11 see extra Excel file with 11 sheets.**

**Table S1**. Basic information of 154 MAGs of the RCA cluster.

**Table S2**. Basic sequencing and genomic information of 87 analysed RCA MAGs/genomes (82 MAGs and 5 genomes from isolates) and locations where the metagenomic samples and isolates were collected.

**Table S3**. Metadata for metagenomic data used to estimate the proportion of the RCA cluster and 10 of the 13 RCA species.

**Table S4**. Metadata for metatranscriptomic data used to estimate the proportion of the RCA cluster and 10 of the 13 RCA species.

**Table S5**: Genome data of Southern Ocean SAG [1].

**Table S6**. Annotation of genes encoded on PGC-containing extrachromosomal replicons of two *Nereidia* strains.

**Table S6**: The incomplete prophage in the *Rhodobacteraceae* bacterium IMCC1909.

**Table S7**: The GC content of genomes and PR genes in the RCA cluster.

**Table S8**: Annotation of genes encoded on PGC-containing extrachromosomal replicons of two *Nereidia* strains.

**Table S9**. List of metabolic pathways, transporters, and genes analyzed in this study.

**Table S10**. Identification of sample origin, location and depth layer and relative abundance of 10 of the 13 RCA species calculated using mOTUs2 results based on metagenomics.

**Table S11**. Identification of sample origin, location and depth layer and relative abundance of 10 of the 13 RCA species calculated using mOTUs2 results based on metatranscriptomics.

**Figure S1**. The variation of the genome size, GC content and CDS number in *Planktomarina* and *Pseudoplanktomarina* species. Values at the top indicate significant *p*-values. The significant difference was tested using the Wilcoxon test using the R package "rstatix" v0.7.2.

**Figure S2**. Phylogenetic tree of southern Ocean SAG [1] and the RCA cluster.

**Figure S3**: Phylogenetic tree of the RCA cluster with two *Nereida* strains as most closely related non-RCA species.

**Figure S4**. Organisation and structure of the PR operon present in genomes of RCA species.. *Blh*: 15,15′-β-carotene dioxygenase; *crt*: carotenoid biosynthesis genes;

S5A

S5B

**Figure S5**. Phylogenetic tree of the *pufM* gene and structure and arrangements of PGC operons detected in the RCA cluster. A: Phylogenetic tree based on 43 *pufM* genes. B: structure and arrangements of PGC operons detected in genomes of *Planktomarina* *C6*, *C7* and *C8*. The tree was constructed using IQ-TREE under the LG+R10 substitution model with 1000 ultrafast bootstraps. Only bootstrap values ≥75 were shown with filled blue circles.

Figure S6A - Metagenomics species A1, B1, B2

Figure S6B – Metagenomics species *B3, B4/5*

Figure S6C – Metagenomics species *C1, C3, C4/C5, C6*

Figure S6D – Metatranscriptomics species *A1*, *B1*, *B2*, *B3*, *B4*

Figure S6E – Metatranscriptomics species *C1*, *C3*, *C4/5, C6*

**Figure S6**: Geographic distribution and relative abundance of ten of the thirteen RCA species (*C1*, *C3*-*C6*, *B1*-*B4*, *A1*) in different layers based on metagenomic and metatranscriptomic data: epipelagic (EPI, 0-200 m), mesopelagic (MES, 200-1000 m) and bathypelagic (BAT >1000 m). The dots in different colours indicated the relative abundance of the species as percent of the RCA cluster.

1. Sun Y, Zhang Y, Hollibaugh JT, Luo H. Ecotype diversification of an abundant Roseobacter lineage. Environ Microbiol. 1625;19:1625–38.
